# Geriatric nutritional risk index as a predictor of prognosis in hepatocellular carcinoma: A systematic review and meta-analysis

**DOI:** 10.12669/pjms.41.4.11962

**Published:** 2025-04

**Authors:** Linfu Fang, Ziwei Chen

**Affiliations:** 1 Linfu Fang Department of Hepatobiliary, Pancreas, Thyroid and Breast Surgery, Sanmen People’s Hospital, Taizhou City, Zhejiang Province 317100, P.R. China; 2 Ziwei Chen Department of Cardiovascular Medicine, Sanmen People’s Hospital, Taizhou City, Zhejiang Province 317100, P.R. China

**Keywords:** Liver cancer, Malnutrition, Prognosis, Recurrence

## Abstract

**Objective:**

The geriatric nutritional risk index (GNRI) has found utility as a predictor of outcomes in several malignancies. However, does it predicts outcomes in hepatocellular cancer (HCC) is unclear. In this review, we present high-quality evidence on the prognostic ability of GNRI for HCC.

**Methods:**

Two reviewers screened the websites of Embase, PubMed, Web of Science, and Scopus up to 20^th^ June 2024 for relevant articles. We examined overall survival (OS) and progression-free survival (PFS) based on low vs high GNRI in HCC.

**Results:**

Total 13 studies were included. Meta-analysis of 11 studies showed that low GNRI was significantly associated with poor OS (HR: 1.83 95% CI: 1.47, 2.29 I^2^=67%) and PFS (HR: 1.51 95% CI: 1.34, 1.69 I^2^=27%.) in HCC patients. No publication bias was noted. Most outcomes did not change on subgroup analysis based on country of origin, sample size, Child-Pugh Grade-B %, treatment, cut-off, follow-up, and method of analysis. Results remained significant on sensitivity analysis.

**Conclusions:**

The GNRI can predict OS and PFS in HCC patients. Given its availability and ease of calculation, the tool can be incorporated into clinical practice to rapidly predict the prognosis of HCC patients.

## INTRODUCTION

Hepatocellular carcinoma (HCC) is the most predominant form of liver cancer seen worldwide accounting for almost 80% of all cases.^1^ Viral diseases, namely hepatitis B & C account for the most important risk factors for HCC, but their importance is on a decline. Improved vaccination coverage and effective treatments for chronic infections have led to a reduction in the incidence of viral-associated HCC.^2^ Nevertheless, this has not decreased the absolute incidence of HCC owing to the increase in metabolic syndrome, obesity, diabetes, and non-alcoholic fatty liver disease which have become one of the most important contributors of HCC.^3^ Early-stage HCC can be managed by surgery, liver transplant, or local therapy like radiofrequency ablation or microwave ablation while chemotherapy, immunotherapy, and targeted therapies are needed for advanced HCC.^4^ Nevertheless, despite advances in diagnostic modalities and treatment protocols, survival after HCC remains poor. Recent data shows that HCC treated by at least one modality has a five-years survival of 45.5% while those not receiving any treatment have a survival of only 9.6%.^5^

Prognostic markers play an important role during counseling, treatment planning, and risk stratification of cancer patients. In recent times, malnutrition has been recognized as an important contributor to worse survival, increased recurrence, and poor treatment response in cancer patients.^6^,^7^ In this context, several nutritional-based markers have been described in the literature. The prognostic nutritional index, controlling nutritional status, geriatric nutritional risk index (GNRI), Malnutrition Universal Screening Tool, Patient-Generated Subjective Global Assessment, Mini-Nutritional Assessment tool, and Nutritional Risk Screening 2002 are some of the tools that have been validated in cancer patients.^8^ȓ^10^ The GNRI is a nutritional assessment tool that was primarily designed for estimating malnutrition in hospitalized elderly patients due to their inability to participate in questionnairebased assessments.^11^ It utilizes simple and easily available measurements namely, albumin and body weight data to stratify a patient as malnourished.

Over the years, the GNRI has been employed to examine the prognosis of several malignancies like gastric,^12^ colorectal,^13^ lung,^14^ and head and neck cancers.^15^ However, its validity in HCC remains unclear. There is only one meta-analysis in the literature that has studied the prognostic ability of GNRI in HCC, albeit with only seven studies.^16^ We hereby present the most updated evidence on the ability of GNRI to predict overall survival (OS) and progression-free survival (PFS) in HCC.

## METHODS

This work adheres to PRISMA^17^ and was registered on PROSPERO (CRD42024559816). We conducted a systematic search of Embase, PubMed, Web of Science, and Scopus on 20^th^ June 2024 to search for studies. We aimed to search peer-reviewed articles without restrictions on the date of publication and language. We supplemented the search with a manual screening of Google Scholar for any missed studies. The search strategy included MeSH and free keywords and was as follows: (liver cancer) OR (hepatocellular carcinoma)) AND ((geriatric nutritional risk index) OR (GNRI))”. The articles found in all repositories were put together and then deduplicated. Initial eligibility was judged by examining the titles and abstracts of the studies. Two investigators were involved in the search and screening process. Studies not pertaining to the review question were eliminated and potentially eligible studies were downloaded for further assessment. The investigators read the full texts and selected eligible articles. We also inspected the references of all studies and prior reviews to identify further studies. Any disagreements were discussed and resolved by consensus.

### Eligibility criteria:

Studies were included in the meta-analysis if they fulfilled the following:

- Applying a case-control or cohort design.
- Conducted on HCC patients.
- Examining the association between GNRI and outcomes.
- Outcomes of interest were OS and PFS.
- Reported the outcome as an effect size with 95% confidence intervals (CI).

Abstracts, reviews, unpublished data, case reports, and duplicate studies were not considered in the review. If the same sample was examined in two articles, the study with the larger sample size was included.

### Quality of studies:

Two reviewers each examined the quality of articles using the Newcastle Ottawa Scale.^18^ A maximum of nine stars can be given to each study. Disagreements between reviewers in the assessment of points were discussed and resolved by consensus.

### Data management:

Relevant data extracted from studies included: the name of the author, database used, location, design, sample size, demographic details, Child-Pugh score, poorly differentiated HCC, treatment, cut-off of GNRI, malnourished as per cut-off, median follow-up, variables adjusted in the analysis, and outcomes. All data was sourced by two authors. The primary outcome was OS and the secondary outcome was PFS.

### Statistical analysis:

Meta-analysis was conducted using Review Manager 5.3 (Cochrane Collaboration, 2014). Effect sizes obtained from the studies were log-transformed and combined in a random-effects model generating pooled hazard ratios (HR) with 95% CI. Heterogeneity among studies was quantified by obtaining the I value and the Chi-square test. P< 0.05 was considered statistically significant and an I^2^ value of 50% or more indicated the presence of heterogeneity. Sensitivity analysis was performed to measure the contribution of each study to the pooled estimate by sequentially excluding individual studies. Visual inspection of asymmetry in funnel plots was conducted to evaluate publication bias. Subgroup analysis was conducted based on country of origin, sample size, Child-Pugh Grade-B %, treatment, cut-off, follow-up, and method of analysis.

## RESULTS

**Fig.1:**
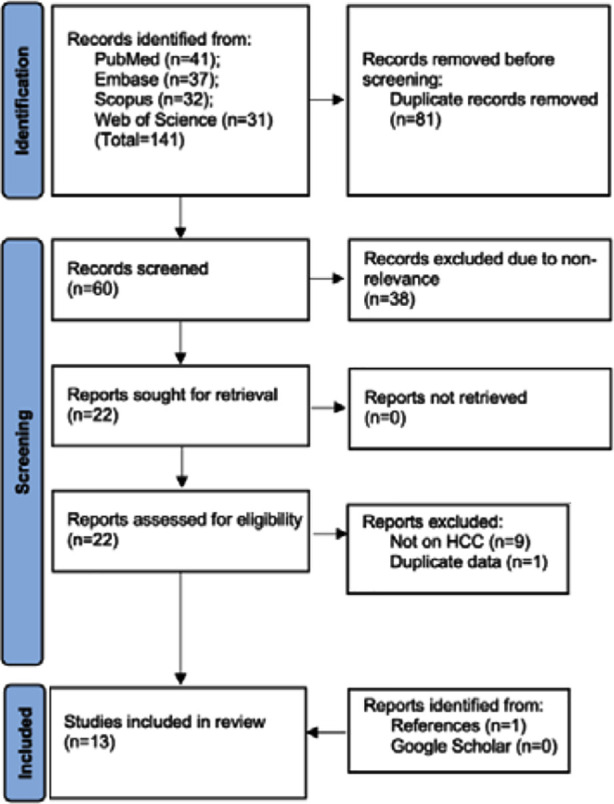
PRISMA flow chart.

The number of results obtained from each database and further steps in the screening process are presented in Fig.1. Total number of articles found were 141.81 duplicates were excluded. Sixty articles were screened by title and abstracts, and 22 were selected for further review. A total of 13 articles were found to be eligible.^13^,^19^–^30^ All studies were published in the past six years. They all were primarily from China and Japan. In total 5405 HCC patients were analyzed by all 13 studies. The median age of included patients was >50 across studies. Male predominance was noted in all studies. All studies had a higher percentage of Child-Pugh class A patients. The percentage of class B ranged from 0-25%. Seven studies were on patients treated with surgery, two were on those treated with transcatheter arterial chemoembolization (TACE) while the remaining studies used immune checkpoint inhibitors (ICI) and/or tyrosine kinase inhibitors (TKI). Most studies used a GNRI cut-off of 98. Two studies used a cut-off of 92 while one used 88.92. Based on this cut-off, the prevalence of malnutrition was 15-51.3%. Two studies reported only univariate analysis while others reported multivariate adjusted data. Adjusted covariates differed across studies. Median follow-up varied from 0.8 to 5.8 years.

### Meta-analysis:

**Fig.2:**
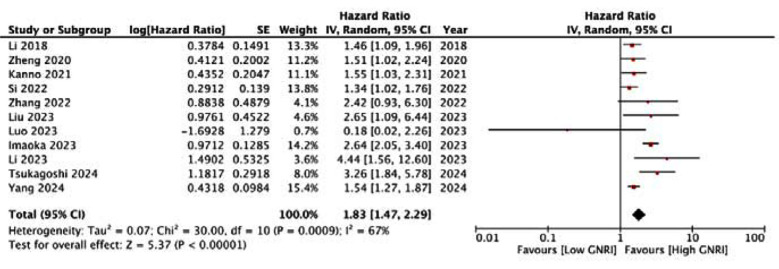
Meta-analysis of OS in HCC with low vs high GNRI.

**Supplementary Fig.1:**
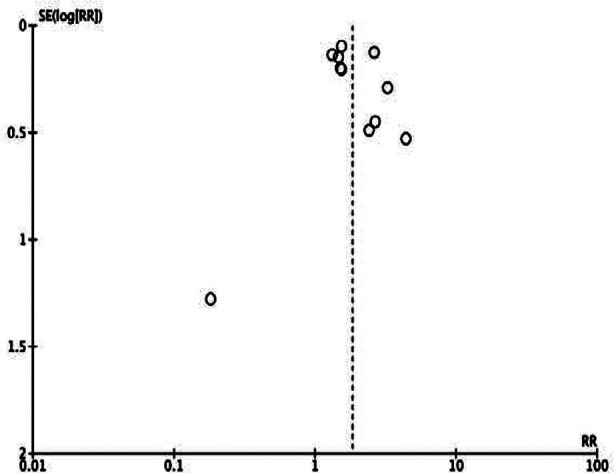
Funnel plot for assessment of publication bias for OS.

**Fig.3:**
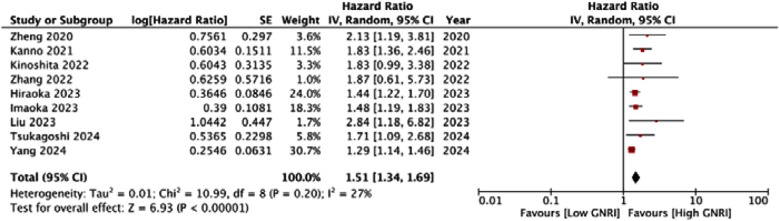
Meta-analysis of PFS in HCC with low vs high GNRI.

**Supplementary Fig.2:**
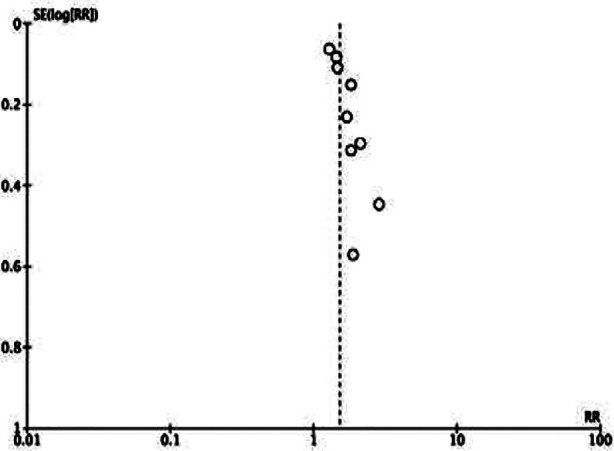
Funnel plot for assessment of publication bias for PFS.

This showed that low GNRI was significantly associated with poor OS in HCC (HR: 1.83 95% CI: 1.47, 2.29) Fig.2. High heterogeneity was seen in the meta-analysis, I^2^=67%. Visual inspection showed no major publication bias (Supplementary Fig.1). Meta-analysis showed that low GNRI was also linked with poor PFS in HCC (HR: 1.51 95% CI: 1.34, 1.69) Fig.3. Low heterogeneity was seen in the meta-analysis, I^2^=27%. Visual inspection showed no major publication bias (Supplementary Fig.2). Supplementary Table-I shows the resultant HR on the singular exclusion of individual studies from the meta-analysis of OS and PFS. No change in significance was noted on the exclusion of any study. During sensitivity analysis, the HR for OS ranged from 1.67 to 1.93 while that for PFS varied from 1.44 to 1.57. The inter-study heterogeneity did not come down to <50% for OS on the exclusion of any study. Heterogeneity for PFS varied from 0-36%.

**Table-I:** Details of included studies.

Study	Location	Design	Sample size	Mean/ Median age	Males (%)	Child-Pugh (%)	Poorly differ-entiated (%)	Treatment	Cut-off	Mal-nourished (%)	Median follow-up (years)	Adjusted variables	NOS score
Li 2018^23^	China	R	261	69	82.4	A: 100	5.7	Surgery	98	24.5	4.8	None	7
Zheng 2020^18^	China	R	154	71	76	A: 81.2 B: 18.8	NR	TACE	92	51.3	5	Age, sex, BMI, ECOG score, Child-Pugh grade, BCLC stage, tumor size, number, PVTT, and AFP	9
Kanno 2021^24^	Japan	R	346	72.5	73.1	A: 96.8 B: 3.2	17	Surgery	98	50	5.8	Age, sex, BMI, DM, Child-Pugh grade, tumor size, number, MVI, preoperative platelet, AFP, and tumor differentiation grade	9
Kinoshita 2022^25^	Japan	R	61	73.1	86.9	NR	NR	Lenvatinib	98	42.6	1.4	Age, sex, Scr, mALBI grade, BCLC stage, portal vein invasion, extrahepatic spread, and AFP	8
Si 2022^26^	China	R	235	59.5	80.9	A: 66.3 B: 33.7	NR	TACE	98	47.2	3	Age, sex, HTN, DM, ascites, BMI, tumor size, number, MVI, AFP, anemia, Child-Pugh grade, and BCLC stage	9
Zhang 2022^19^	China	R	85	70	52.9	NR	NR	Surgery	98	17.6	3.4	Age, sex, tumor size, number, and Child-Pugh grade	9
Hiraoka 2023^27^	Japan	R	525	74	80	A: 92.2 B: 7.5 C: 0.2	NR	Atezolizumab plus bevacizumab	98	46.1	0.8	Age, sex, etiology, liver cirrhosis, AFP, BCLC, Child–Pugh class	9
Imaoka 2023^28^	Japan	R	1494	70.4	77.8	A: 92.8 B: 2.7	NR	Surgery	92	15	5	Age, sex, tumor size, number, vascular invasion, Child-Pugh grade, AFP, surgery time, and bleeding volume	9
Li 2023^29^	China	R	100	68.8	70	A: 84 B: 16	NR	Surgery	98	20	1	Age, sex, hepatitis B markers, differentiation, tumor diameter, number of tumors, ascites, lymph node metastasis, TNM stage, envelop integrity, PVTT, Child–Pugh class, AFP	8
Liu 2023^20^	China	R	151	57.4	82.1	NR	NR	ICI	88.92	17.9	NR	Sex, surgery, tumor number, tumor size, cirrhosis, BCLC, TNM stage, inflammatory markers	8
Luo 2023^21^	China	R	124	53.5	100	A: 75 B: 25	NR	ICI and TKI	98	50.8	1.6	Alanine aminotransferase, aspartate aminotransferase, total bilirubin, direct bilirubin, hemoglobin, white blood cells, lymphocyte count, blood urea nitrogen, Child-Pugh grade and China liver cancer staging	8
Tsukagoshi 2024^22^	Japan	R	203	71.5	81.8	A: 97 B: 3	17.7	Surgery	98	17	3.3	None	7
Yang 2024^30^	China	R	1666	NR	85.2	A: 96.2 B: 3.8	NR	Surgery	98	37.7	NR	Age, sex BCLC stages, liver cirrhosis and portal hypertension	8

GNRI, Geriatric nutritional risk index; R, Retrospective cohort; HCC, Hepatocellular carcinoma; TACE, Transcatheter arterial chemoembolization; MVI, Microvascular invasion; AFP, Alpha fetoprotein; PVTT, Portal vein tumor thrombus; ECOG, Eastern Cooperative Oncology Group; BCLC, Barcelona clinic liver cancer; BMI, Body mass index; DM, Diabetes mellitus; HTN, Hypertension; SCr, Serum creatinine; mALBI, Modified albumin-bilirubin; OS, Overall survival; PFS, Progression-free survival; ICI, immune checkpoint inhibitors; TKI, tyrosine kinase inhibitors

**Supplementary Table-I:** Results of sensitivity analysis.

Overall survival			Progression free survival		
Study removed	Obtained Risk ratio [95% confidence interval]	I^2^	Study removed	Obtained Risk ratio [95% confidence interval]	I^2^
Li 2018^23^	1.91 [1.48, 2.45]	68	Zheng 2020^18^	1.48 [1.32, 1.65]	24
Zheng 2020^18^	1.89 [1.48, 2.41]	70	Kanno 2021^24^	1.44 [1.30, 1.61]	15
Kanno 2021^24^	1.88 [1.47, 2.40]	70	Kinoshita 2022^25^	1.50 [1.33, 1.70]	33
Si 2022^26^	1.93 [1.51, 2.46]	65	Zhang 2022^19^	1.51 [1.34, 1.71]	35
Zhang 2022^19^	1.81 [1.44, 2.28]	70	Hiraoka 2023^27^	1.57 [1.35, 1.84]	36
Imaoka 2023^28^	1.67 [1.38, 2.03]	46	Imaoka 2023^28^	1.55 [1.34, 1.79]	36
Li 2023^29^	1.77 [1.42, 2.20]	66	Liu 2023^20^	1.47 [1.32, 1.63]	19
Liu 2023^20^	1.80 [1.43, 2.26]	69	Tsukagoshi 2024^22^	1.50 [1.33, 1.70]	33
Luo 2023^21^	1.86 [1.50, 2.30]	67	Yang 2024^30^	1.57 [1.40, 1.75]	0
Tsukagoshi 2024^22^	1.74 [1.40, 2.16]	64			
Yang 2024^30^	1.91 [1.46, 2.49]	68			

### Subgroup analysis:

The outcomes of subgroup analyses can be found in Supplementary Table-II. In the case of OS, subgroup analysis based on country (China, Japan), sample size (>250, <250), and cut-off (98, 92, 88.92) did not alter the significance of HR. Results turned non-significant in studies with Child-Pugh Grade-B <10%, treatment with ICI and TKI, follow-up <3 years, and those with only univariate analysis. The results of PFS remained stable on subgroup analysis and did not turn non-significant on any subgroup analysis.

**Supplementary Table-II:** Subgroup analysis results.

Variable	Groups	Studies	Risk ratio [95% confidence intervals]	I^2^
Overall survival				
Country	China	8	1.55 [1.30, 1.86]	31
	Japan	3	2.33 [1.56, 3.48]	68
Sample size	>250	4	1.75 [1.31, 2.35]	78
	<250	7	2.00 [1.34, 2.99]	63
Child Pugh Grade-B	>10%	5	1.90 [1.42, 2.55]	78
	<10%	4	1.56 [0.98, 2.50]	59
Treatment	Surgery	7	2.02 [1.53, 2.67]	72
	ICI & TKI	2	0.91 [0.07, 11.85]	74
	TACE	2	1.39 [1.11, 1.74]	0
Cut-off	98	8	1.68 [1.34, 2.11]	54
	92	2	2.04 [1.18, 3.52]	82
	88.9	1	2.65 [1.09, 6.44]	-
Follow-up	≥3 years	6	1.95 [1.46, 2.61]	68
	<3 years	3	1.53 [0.48, 4.85]	73
Analysis	Multivariate	9	1.80 [1.40, 2.31]	67
	Univariate	2	2.10 [0.96, 4.59]	83
Progression free survival				
Country	China	4	1.71 [1.14, 2.56]	50
	Japan	5	1.53 [1.37, 1.72]	0
Sample size	>250	5	1.44 [1.29, 1.62]	32
	<250	4	2.09 [1.46, 3.00]	0
Child Pugh Grade-B	>10%	1	2.13 [1.19, 3.81]	-
	<10%	5	1.44 [1.29, 1.62]	32
Treatment	Surgery	5	1.47 [1.27, 1.71]	34
	ICI & TKI	3	1.61 [1.20, 2.16]	25
	TACE	1	2.13 [1.19, 3.81]	-
Cut-off	98	6	1.46 [1.29, 1.65]	25
	92	2	1.60 [1.19, 2.14]	25
	88.9	1	2.84 [1.18, 6.82]	-
Follow-up	≥3 years	5	1.64 [1.41, 1.91]	0
	<3 years	2	1.46 [1.25, 1.72]	0
Analysis	Multivariate	8	1.50 [1.33, 1.70]	33
	Univariate	1	1.71 [1.09, 2.68]	-

ICI, immune checkpoint inhibitors; TKI, tyrosine kinase inhibitors; TACE, Transcatheter arterial chemoembolization.

### Risk of bias analysis:

Quality assessment of studies is presented in Supplementary Table-III. Six studies received a NOS score of nine indicating high quality. Two studies received a score of seven while the remaining got a score of eight.

**Supplementary Table III:** Details of risk of bias analysis.

Study	Selection of cohort	Comparability	Outcome assessment	NOS score
Li 2018^23^	4	-	3	7
Zheng 2020^18^	4	2	3	9
Kanno 2021^24^	4	2	3	9
Kinoshita 2022^25^	4	2	2	8
Si 2022^26^	4	2	3	9
Zhang 2022^19^	4	2	3	9
Hiraoka 2023^27^	4	2	3	9
Imaoka 2023^28^	4	2	3	9
Li 2023^29^	4	2	2	8
Liu 2023^20^	4	2	2	8
Luo 2023^21^	4	2	2	8
Tsukagoshi 2024^22^	4	-	3	7
Yang 2024^30^	4	2	2	8

NOS, Newcastle Ottawa scale.

## DISCUSSION

This updated systematic review and meta-analysis examined the association between GNRI OS and PFS in patients with HCC. Thirteen studies with 5405 HCC patients of different stages undergoing different treatments were included in the meta-analysis which showed that patients with low GNRI scores had worse OS and PFS as compared to those with normal GNRI. Low GNRI led to an 83% increase in the risk of poor OS ranging from 47% to 129% and a 51% increase in the risk of poor PFS ranging from 34% to 69%. Similar results have been obtained by Xu et al.^16^ in the prior meta-analysis wherein they examined evidence from seven studies and found that GNRI was a predictor of poor OS (HR: 1.77, 95 % CI: 1.32, 2.37) and PFS (HR: 1.62 95% CI: 1.39, 1.89) in HCC. The current review is a significant update over this review as we included six more studies thereby improving the statistical power of the evidence.

Our results are also in agreement with prior meta-analysis studies which have reviewed evidence on the prognostic ability of GNRI. Including eight studies with 2399 patients, Wang et al.^31^ in their review have recognized that GNRI can predict OS, recurrence-free survival, and cancer-specific survival in non-small cell lung cancer patients. Mao et al.^32^ examined data from ten cohort studies including 3802 colorectal cancer patients undergoing surgery and noted that low GNRI was predictive of OS and disease-free survival. Similar results were replicated by Yiu et al.^33^ in their meta-analysis of ten studies and 2793 patients which showed that low GNRI was associated with worse OS in head and neck cancer patients. Li et al.^34^ combined data from six studies with 1513 patients to show a positive association between low GNRI and poor OS but not PFS in pancreatic cancer.

Another recent review by Yu et al.^35^ including 14 patients with 3524 patients corroborated the prognostic ability of GNRI in hematological malignancies. Given such vast evidence on GNRI, it can be inferred that GNRI is a reliable prognostic marker for cancer patients but it requires validation for different cancer subtypes. Cancer survival shows large heterogeneity based on its tissue of origin ranging from just 1.1% for pancreatic cancer to up to 98% for testicular cancer.^36^ Through the present review, we have extended the validity of GNRI to HCC thereby providing a simple, readily available, objective, and easy-to-use tool for clinicians involved in the management of such patients. An area of concern in the interpretation of current evidence is high heterogeneity in the meta-analysis, especially of OS. It is pertinent to note that the included studies had baseline methodological heterogeneity due to differences in the studied population. None of the studies segregated data based on the etiology of the disease (viral vs alcoholic vs metabolic), stage of HCC (early vs advanced), histopathology (well-differentiated vs moderately differentiated vs poorly differentiated), and first-line treatment undertaken.

A wide class of HCC patients were examined in the included cohorts thereby generating high inter-study heterogeneity. Nevertheless, attempts were made to identify its source by conducting a thorough sensitivity and subgroup analysis. Sensitivity analysis did not find any outlier study in the meta-analysis as the significance of the HR did not change for both outcomes. Also, there was no reduction in the heterogeneity of OS on sequential exclusion of studies. A detailed subgroup analysis based on country of origin, sample size, Child-Pugh Grade-B %, treatment, cut-off, follow-up, and method of analysis was also undertaken which showed some non-significant results in the case of OS but not PFS indicating that evidence is robust for the latter. For OS, non-significant results were seen for subgroups involving Child-Pugh Grade-B <10%, treatment with ICI and TKI, follow-up <3 years, and those with only univariate analysis. Most of these subgroups included very few studies and except for the subgroup of treatment with ICI and TKI, all others had an overall HR of >1. This shows that the statistical power of these subgroups may have not been high enough for significant results.

The high validity of GNRI in predicting outcomes of HCC as well as other cancers has been ascribed to its two components i.e. albumin and body weight. Albumin levels directly reflect the baseline nutritional status of the patient.^37^ Furthermore, albumin is also a marker of systemic inflammatory response in cancer and has been singularly associated with poor cancer-related survival.^38^ As albumin is primarily produced by the liver it also indicates baseline liver function of the individual. Hypoalbuminemia has been found to predict worse OS, PFS, increased complications, and poor chemotherapy response in HCC.^39^ȓ^41^ Normal albumin levels are also needed to decrease the spread of HCC. Albumin reduces the phosphorylation of retinoblastoma protein and increases expression of p21 and p57 which causes the proliferation of G0/G1 cell population in the liver thereby slowing the spread of HCC.^42^ An additional function of albumin is immunomodulation which causes macrophage activation and triggers cell-mediated immunity against cancer cells.^7^ The second component of GNRI is body weight which is calculated as the ratio of current body weight to ideal body weight. This incorporates body mass index into the tool which is known to predict survival after HCC.^43^

### Limitations

Firstly, high heterogeneity was noted in the meta-analysis of OS which failed to diminish on sensitivity and subgroup analyses. We believe that such high heterogeneity originated from differences in study populations, cancer stage, and treatment protocols utilized by the included studies. Since our review was a study-level meta-analysis and not a patient-level meta-analysis, we could not perform a thorough meta-regression for such variables. Secondly, there was some variability in GNRI cut-offs used by the included cohorts. Difference in cut-offs directly impacts the prevalence of malnutrition and hence future studies must use similar cut-offs to produce robust results. Thirdly, all data originated from just two countries. The lack of data from other regions diminishes the validity of GNRI in the global population. Since HCC is a global healthcare problem, the reason for such disparity is unclear. More studies from other countries should report the prognostic role of GNRI in HCC for more robust evidence. Fourthly, while most studies reported adjusted data the included confounders differed. It is quite possible that missed or unknown variables could have influenced outcomes. Lastly, all included studies examined pretreatment GNRI scores. There was no study examining the impact of change in GNRI scores on prognosis. There is scope for future research examining if nutritional support during HCC therapy alters GNRI and thereby outcomes.

## CONCLUSION

The GNRI can predict OS and PFS in HCC patients. Given its availability and ease of calculation, the tool can be incorporated into clinical practice to rapidly predict the prognosis of HCC patients. Further research validating GNRI for different HCC stages and treatments is needed from different regions of the world.

### Authors’ contributions:

**LF and ZC:** Literature search, study design and manuscript writing.

**LF and ZC:** Data collection, data analysis and interpretation Critical review.

**LF:** Literature search, manuscript revision and validation.

All authors have read, approved the final manuscript. They are also responsible for the integrity of the study.
